# O09 - Early supplementation with Lactobacillus rhamnosus HN001 reduces eczema prevalence to 6 years: does it also reduce atopic sensitisation?

**DOI:** 10.1186/2045-7022-4-S1-O9

**Published:** 2014-03-14

**Authors:** Kristin Wickens, Thorsten Stanley, Edwin Mitchell, Christine Barthow, Penny Fitzharris, Gordon Purdie, Robert Siebers, Peter Black, Julian Crane

**Affiliations:** 1 University of Otago, Wellington, New Zealand; 2 University of Auckland, Auckland, New Zealand; 3 Auckland Hospital, Auckland, New Zealand

## Background

The role of probiotics in prevention of allergic disease is still unclear; efficacy may depend on the timing, dose, duration and specific probiotic used. Using a double blind randomized placebo-controlled trial (Australian New Zealand Clinical Trials Registry: ACTRN12607000518460), we have shown that in a high risk birth cohort, maternal supplementation from 35 weeks gestation until 6 months if breastfeeding and infant supplementation from birth until 2 years with *Lactobacillus rhamnosus* HN001 (HN001) (6 x 10^9^ cfu/day) halved the cumulative prevalence of eczema at 2 and 4 years. *Bifidobacterium animalis* subsp *lactis* HN019 (HN019) (9 x 10^9^/cfu day) had no significant effect.

## Objective

To determine whether differences in effects of HN001 and HN019 on eczema persist to age 6 years, and to investigate effects on sensitization.

## Methods

Standard procedures were used to assess eczema (The UK Working Party’s criteria), eczema severity (SCORAD), atopic sensitization (skin prick tests (SPT), total and specific IgE) and standard questions used for asthma, wheeze and rhinoconjunctivitis.

## Results

HN001 was associated with significantly lower cumulative prevalence of eczema (HR=0.56, 95% CI 0.39-0.80), SCORAD≥10 (HR=0.69, 0.49-0.98) and SPT sensitization (HR=0.69, 95% CI 0.48-0.99). The point prevalence of eczema (RR=0.66, 95% CI 0.44-1.00), SCORAD≥10 (RR=0.62, 95% CI 0.38-1.01) and SPT sensitization (RR=0.72, 95% CI 0.53-1.00) were also reduced among children taking HN001. HN019 had no significant effect on any outcome.

## Conclusion and clinical relevance

This study provides evidence for the efficacy of the probiotic *Lactobacillus rhamnosus* HN001 in preventing the development of eczema and possibly also atopic sensitization in high risk infants to age 6 years. The absence of a similar effect for HN019 indicates that benefits may be species-specific.

